# On Preparing Acetate of Potash, so as to Obtain It in a Saturated State, and of a White Colour

**Published:** 1811-06

**Authors:** 


### *On preparing Acetate of Potash, so as to obtain it in a saturated State, and of a White Colour. By Messrs.* Bernoully *and* Fremy.

(Annales de Chimie.)

Mr. Bernoully, of Basil, commenced his memoir with attempting to discover the colouring matter that is so injurious to the appearance of the salt, and which does not belong either to the potash or the acetic acid, so that it must be a foreign matter contained in common vinegar, and carried over with it in distillation. This principle is less volatile than acetic acid, as distilled vinegar leaves a residuum on being evaporated. It is not very soluble itself without the assistance of the acid, as it is partly precipitated when the acid is saturated with potash. It is evidently of a vegeto-animal nature, from the odour that it exhales upon a burning coal, and from the prussiate of ammonia that is yielded in the distillation of acetate of potash prepared with distilled vinegar, but which does not occur in the distillation of that prepared with radical vinegar. So that the colouring matter appears to be some remains of the yeast left in common vinegar, vinegar, carried over when it is distilled, and more or less altered by this operation.

The acetate of potash may also be discoloured by the empyreumatic oil with which the distilled vinegar becomes charged when the operation is pushed too far. The accidental sullying arising from the oxyds of iron and of manganese contained in the common alkalies, or from metallic vessels being employed in its preparation, is easily avoided.

In order to get rid of the yeast, it is necessary to choose for distillation clear, very strong, and completely fermented vinegar, and to distill with a very gentle heat, not exceeding that of a gentle boiling heat. If, however, in spite of these precautions, the acetate should not be white, the solution must be gently boiled with well-calcined charcoal previous to evaporation; the operation must be conducted slowly.

The distillation of the vinegar must be stopped the instant that the empyreumatic oil begins to come over: for although the succeeding vinegar may be perfectly white, it becomes coloured during the evaporation, and when this once happens it cannot be remedied.

Mr. Fremy's memoir enters into more details. In crystallising the acetate, it is extremely difficult to separate the crystals from the mother-water; he therefore tried the effects of double decompositions. Acetate of lime treated with carbonate or sulphate of potash afforded a salt of the usual colour. Common acetate of lead and carbonate of soda afforded a salt sufficiently white; but an error in the quantities of the ingredients may be attended with bad consequences. He found that the colour of the acetate proceeds not only from some foreign colouring substance that is contained in the distilled vinegar, but also from the alkali itself when it is in excess, and thus enabled to re-act upon the foreign substance; for on preparing two portions of acetate with the same distilled vinegar, one of which had designedly an excess of acid, and the other of alkali, the latter portion was evidently deeper coloured than the former.

To destroy the colouring matter, he filtered the distilled vinegar upon charcoal, then nearly saturated it with carbonate of potash, and carefully retained an excess of acid during the evaporation, by which means he obtained an acetate as white as if it had been melted.

Although this process is very simple, it is not practicable, because the acetate of potash is mixed with acetate of lime coming from the charcoal, which is prejudicial to the drying. It would be easy to separate the lime, by adding a slight excess of carbonate of potash, afterwards adding an excess of the acid; but it is more simple to saturate the acid at at first. The liquor being left upon the charcoal for twenty days, and exposed to the sun's rays, furnished a salt still whiter than before; and Mr. Fermy imagines the same effect may be obtained by exposure to the rays of the sun without the addition of charcoal.

Part of the colouring matter is precipitated during the saturation of the vinegar: it is but slightly soluble in water, but some portion of it remains in the solution of the acetate of potash. When distilled vinegar has been filtrated upon very pure charcoal, such as that from sugarcandy, the same precipitate is not obtained as before the filtration.

Two hecatogrammes (6 oz.) of excellent acetic ether were obtained by rectifying the first produce of the distillation of 70 litres (quarts) of distilled vinegar upon potash.

The discoloration of the acetate by exposing it to the solar rays, did not succeed when tried by the Committee to whom the two preceding memoirs were referred.

